# Identification of Fromiamycalin and Halaminol A from Australian Marine Sponge Extracts with Anthelmintic Activity against *Haemonchus contortus*

**DOI:** 10.3390/md17110598

**Published:** 2019-10-23

**Authors:** H. M. P. Dilrukshi Herath, Sarah Preston, Abdul Jabbar, Jose Garcia-Bustos, Aya C. Taki, Russell S. Addison, Sasha Hayes, Karren D. Beattie, Sean L. McGee, Sheree D. Martin, Merrick G. Ekins, John N. A. Hooper, Bill C. H. Chang, Andreas Hofmann, Rohan A. Davis, Robin B. Gasser

**Affiliations:** 1 Faculty of Veterinary and Agricultural Sciences, The University of Melbourne, Parkville, Victoria 3010, Australia; herathh@student.unimelb.edu.au (H.M.P.D.H.); sjpreston@federation.edu.au (S.P.); jabbara@unimelb.edu.au (A.J.); Jose.GarciaB@unimelb.edu.au (J.G.-B.); aya.taki@unimelb.edu.au (A.C.T.); bchang@yourgene.com.tw (B.C.H.C.); a.hofmann@structuralchemistry.org (A.H.); 2 Faculty of Health and Life Sciences, Federation University, Ballarat, Victoria 3350, Australia; 3 Griffith Institute for Drug Discovery, Griffith University, Brisbane, QLD 4111, Australia; r.addison@griffith.edu.au (R.S.A.); sasha.hayes@griffith.edu.au (S.H.); k.beattie@griffith.edu.au (K.D.B.); 4 Metabolic Research Unit, Metabolic Reprogramming Laboratory, School of Medicine, Faculty of Health, Deakin University, Waurn Ponds, Victoria 3216, Australia; sean.mcgee@deakin.edu.au (S.L.M.); sheree.martin@deakin.edu.au (S.D.M.); 5 Queensland Museum, South Brisbane BC, QLD 4101, Australia; merrick.ekins@qm.qld.gov.au (M.G.E.); john.hooper@qm.qld.gov.au (J.N.A.H.)

**Keywords:** marine, anthelmintic, *Haemonchus contortus*, alkaloid, fromiamycalin, halaminol A, *Monanchora unguiculata*, Haliclona sp

## Abstract

There is an urgent need to discover and develop new anthelmintics for the treatment of parasitic nematodes of veterinary importance to circumvent challenges linked to drug resistant parasites. Being one of the most diverse natural ecosystems, the marine environment represents a rich resource of novel chemical entities. This study investigated 2000 extracts from marine invertebrates, collected from Australian waters, for anthelmintic activity. Using a well-established in vitro bioassay, these extracts were screened for nematocidal activity against *Haemonchus contortus* — a socioeconomically important parasitic nematode of livestock animals. Extracts (designated *Mu*-1, *Ha*-1 and *Ha*-2) from two marine sponges (*Monanchora unguiculata* and *Haliclona* sp.) each significantly affected larvae of *H. contortus*. Individual extracts displayed a dose-dependent inhibition of both the motility of exsheathed third-stage larvae (xL3s) and the development of xL3s to fourth-stage larvae (L4s). Active fractions in each of the three extracts were identified using bioassay-guided fractionation. From the active fractions from *Monanchora unguiculata*, a known pentacyclic guanidine alkaloid, fromiamycalin (**1**), was purified. This alkaloid was shown to be a moderately potent inhibitor of L4 development (half-maximum inhibitory concentration (IC_50_) = 26.6 ± 0.74 µM) and L4 motility (IC_50_ = 39.4 ± 4.83 µM), although it had a relatively low potency at inhibiting of xL3 motility (IC_50_ ≥ 100 µM). Investigation of the active fractions from the two *Haliclona* collections led to identification of a mixture of amino alcohol lipids, and, subsequently, a known natural product halaminol A (**5**). Anthelmintic profiling showed that **5** had limited potency at inhibiting larval development and motility. These data indicate that fromiamycalin, other related pentacyclic guanidine alkaloids and/or halaminols could have potential as anthelmintics following future medicinal chemistry efforts.

## 1. Introduction

The oceans harbour a highly diverse array of fauna and flora, making them one of the most attractive systems for the exploration of novel chemical entities [1,2,3]. Marine organisms produce secondary metabolites as chemical ‘defences’ against various types of pressures in the environment, such as predators, parasites and/or competitors in the habitat space [4,5]. This evolutionary process has led to the production of numerous natural compounds with unique chemical structures and a range of biological activities that allow for therapeutic applications [5], including vidarabine (*ara*-A; an anti-viral agent), ω-conotoxin MVIIA ziconotide (a pain control agent), ω-3 acid ethyl esters (serum triglyceride reducing agent) and anticancer agents, including cytarabine (*ara*-C), trabectedin (ecteinascidin 743), eribulin mesylate and brentuximab vedotin, which have been approved by the US-Food and Drug Administration (FDA) and commercialised [6].

Although the marine ecosystem has a rich biodiversity, most of it has not yet been explored [7]. Therefore, compounds produced by known or unclassified marine species may not have been studied for biological activities. The main challenges of systematic investigations of marine samples include difficulties and costs associated with collecting materials, establishing, curating and maintaining large-scale collections; the culturing of marine organisms to produce large amounts of biologically-active compounds; and, in general, constraints with the supply of these precious resources for screening and subsequent testing in biological assays [7]. However, substantial efforts have been made to build and maintain collections of marine samples. For instance, institutes including PharmaMar, Madrid, Spain (https://www.pharmamar.com/), the Australian Institute for Marine Science (AIMS; https://www.aims.gov.au/), Griffith Institute for Drug Discovery (GRIDD) in Queensland, Australia (https://www.griffith.edu.au/institute-drug-discovery), and the National Cancer Institute of the National Institutes of Health, USA [8], have maintained and curated substantive collections of marine materials as well as extracts and fractions for a range of research purposes, including drug discovery.

Given that Australia has the third largest marine jurisdiction in the world and is recognised as a “hot spot” for marine biodiversity [9,10], it serves as an excellent geographic region for the discovery of new chemical entities from nature. In the last decade, Australian oceans have gifted a range of bioactive compounds, including some with activity against the parasitic nematode *Haemonchus contortus* [11,12]. These compounds include geodin A [13], onnamide F [14], thiocyanatins 1–4 [15] and echinobetains A and B [16,17]. All of these compounds were purified from the marine specimens collected from the southern oceanic zone of Australia. However, the frequency of regulatory approval of new anthelmintics is low, and there has been no new anthelmintic released for use in livestock animals since derquantel, which reached the market in 2010 [18]. Therefore, there is a need to explore and develop novel anthelmintic candidates to circumvent the widespread drug resistance problem that exists in populations of parasitic roundworms (nematodes) infecting livestock animals around the world [19,20,21].

As an initial step in exploring and developing anthelmintic compounds from the Australian marine environment, we sourced a marine extract collection from NatureBank at GRIDD, which maintains extract libraries from a range of marine organisms, primarily invertebrates, collected from waters off the coasts of Queensland (including the Great Barrier Reef) and Tasmania. We screened a subset of 2000 extracts from this library for anthelmintic activity and undertook bioassay-guided fractionation to purify the compounds with activity against *H. contortus*.

## 2. Results and Discussion

### 2.1. Three Extracts from Marine Sponges Exhibited Anthelmintic Activity Against Larval Stages of H. contortus 

The screening of 2000 lead-like enhanced (LLE) marine extracts [22] identified only three extracts from marine sponges that reduced exsheathed third-stage larvae (xL3) motility by ≥70%. *Mu*-1 was an extract from *Monanchora unguiculata* (family Crambeidae), collected from a coral wall on the Gibson Reef, Queensland, at a depth of 22.4 m on 26 November 2004 (voucher specimen QM G321891, Queensland Museum, Brisbane, Australia). *Ha*-1 was an extract from the sponge *Haliclona* sp. (family Chalinidae), collected from Wistari Point, Heron Island Coral Reef, Queensland, Australia, at a depth of 17.6 m on 4 November 2009 (voucher specimen QMG321606). *Ha*-2 was also an extract from *Haliclona* sp., sourced from the North-West Ledge, Heron Island Reef, Queensland, at a depth of 30.2 m on 4 November 2010 (voucher specimen QM G321667). The sponges were collected, identified, prepared, issued voucher specimen (QM) codes and curated by colleagues from the Queensland Museum, Brisbane, Australia.

**Figure 1:**
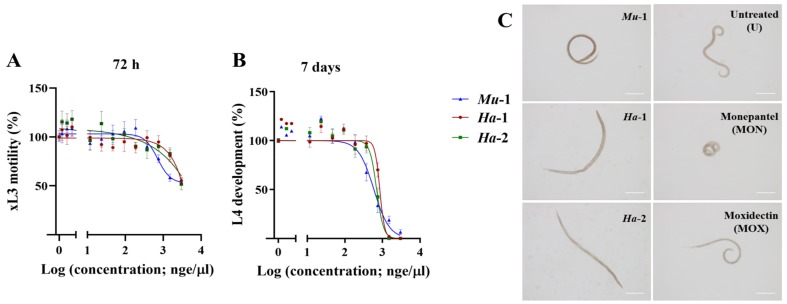
In vitro activity of extracts on the inhibition of xL3 motility and/or L4 development of *Haemonchus contortus*. Dose-response curves for the extract (*Mu-*1) from *Monanchora unguiculata*, and two extracts (*Ha*-1 and *Ha*-2) from *Haliclona* sp. from two geographic locations, for the inhibition of xL3 motility (panel **A**) and of L4 development (panel **B**). Representative light microscopy images of xL3s treated with extract *Mu*-1 showing a ‘circular’ phenotype, and ‘straight’ phenotype of xL3s treated separately with *Ha*-1 and *Ha*-2, compared with unaffected larvae in the untreated (U) control, and ‘coiled’ and ‘circular’ xL3s treated with monepantel (MON) and moxidectin (MOX), respectively. White scale bar: 100 µm; 20x magnification (panel **C**).

Each of these three extracts (i.e., *Mu*-1, *Ha*-1 and *Ha*-2) exhibited a dose-dependent inhibition of xL3 motility (half-maximum inhibitory concentration (IC_50_) values ranging from 0.7 ± 0.01 µge/µL to ~ 3.0 µge/µL; see Section 3.1 for an explanation of “µge/µL”) and of fourth-stage larvae (L4) development (IC_50_ values of ~ 0.6 µge/µL for each extract) of *H. contortus* (Figure 1 and Table 1). The *Mu*-1-treated xL3s exhibited a ‘circular’ phenotype, whereas xL3s treated separately with extracts *Ha*-1 and *Ha*-2 showed a ‘straight’ phenotype (Figure 1).

Interestingly, all three active extracts investigated here originated from demosponges (i.e., species of *Monanchora* and *Haliclona*). Sponges/demosponges and/or their associated micro-organisms account for most of the marine-derived natural compounds isolated thus far [6]. The extracts and compounds purified from *Monanchora* and *Haliclona* have previously been reported to possess a range of biological activities. For instance, bioactive compounds from the genus *Monanchora* include crambescidins and unguiculin A–C (anti-cancer) [23,24,25], ptilomycalin F (anti-malarial) [26], batzelladines (anti-protistan) [27], dehydrocrambine (anti-viral) [28] and merobatzelladines A and B (anti-bacterial) [29]. Extracts from *Haliclona* have been reported to exhibit anti-bacterial [30], anti-fungal [30], anti-filarial [31,32] and anti-*Leishmania* [33] activities. Moreover, compounds with anti-cancer activity (e.g., brominated acetylenic hydrocarbons and halilectin-3) [34,35] as well as anti-infective candidates, including haliclocyclamines A–C (anti-mycobacterial) [36] and steroid compounds (anti-*Trypanosoma*) [37], have been purified from the genus *Haliclona*. However, activity against animal-parasitic nematodes has not been reported previously for extracts from either of these two marine sponge species. 

### 2.2. Bioassay Screening Identified Active Chromatographic Fractions from Three Marine Sponge Extracts

**Figure 2:**
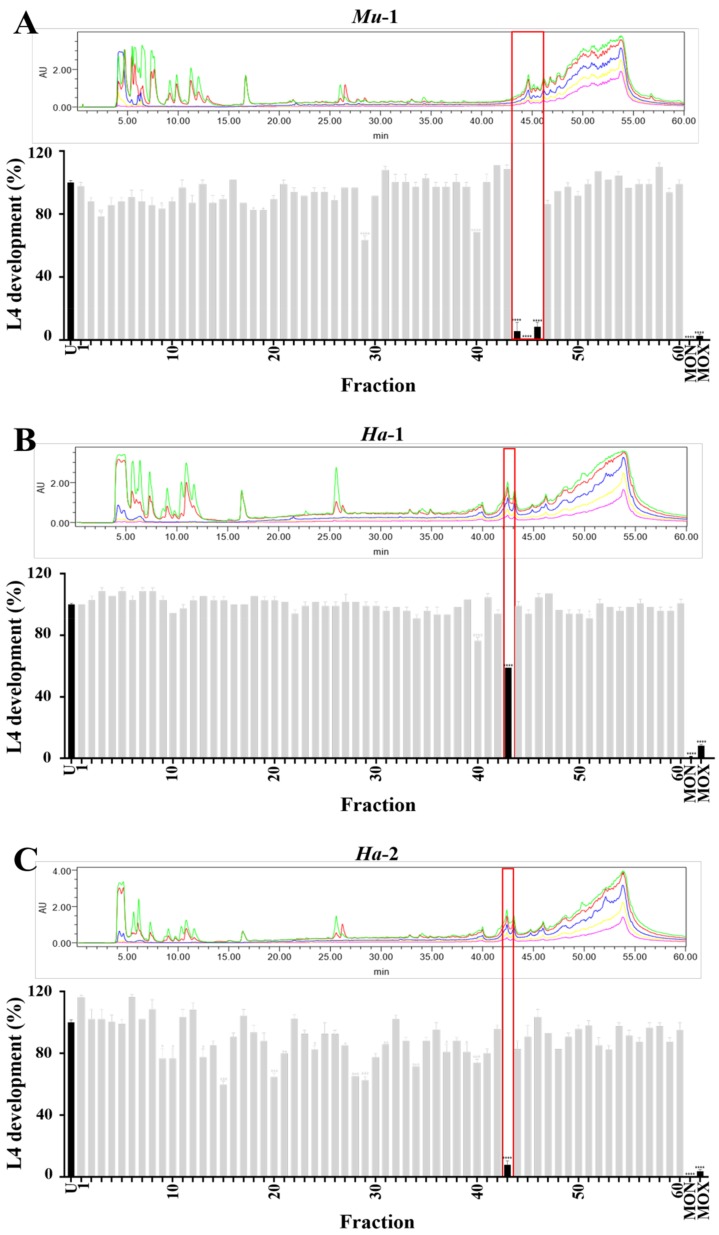
The chromatograms and L4 development (%) for C_18_ semi-preparative fractions of the three active extracts compared with that of control compounds; untreated (U, LB* + 1.25% DMSO) and positive controls MON and MOX. For extract *Mu*-1, fractions 44–46 elicited the most significant inhibition (panel **A**), whereas fraction 43 elicited the highest and significant inhibition for extracts *Ha*-1 and *Ha*-2 (panels **B** and **C**). The five individual chromatograms in panels **A**–**C** display the ultraviolet (UV)-absorbance of the eluting fractions at 254 nm (green), 280 nm (red), 320 nm (blue), 350 nm (yellow) and 380 nm (pink). Fraction/s with the highest L4 developmental inhibition are indicated by red rectangles in panels **A**–**C**. ****P < 0.0001, ***P < 0.001, **P < 0.01, *P < 0.05.

The chromatographic fractions (n = 60) from each active lead-like enhanced (LLE) extract (i.e., *Mu*-1, *Ha*-1 and *Ha*-2) were tested for inhibitory effects on xL3 motility and L4 development. None of the fractions (from any LLE extract) inhibited xL3 motility by ≥ 70%. These observed differences in xL3 motility inhibition between extracts and their respective fractions might be due to the relatively high concentrations of active compounds present in the extracts and/or the synergistic effects of the compounds. However, for *Mu*-1, three consecutive fractions (44–46), which corresponded to a singular ultraviolet (UV)-absorbance peak in the HPLC chromatogram, elicited a pronounced inhibition of L4 development (Figure 2). In addition, fraction-43 elicited the most significant (*P* < 0.0001) reduction in L4 development of both extracts *Ha*-1 and *Ha*-2 (Figure 2). The observed differences in the degree of reduction of L4 development by the active fractions of these two extracts (see Figure 2) might be associated with differences in the mixture of active compound present (see Section 2.5), resulting from the variations in location or time of collection and/or genetic variability of the sponge and/or its associated micro-organisms.

### 2.3. NMR, MS and Chiro-optical Data Analyses Identified Fromiamycalin in Active Fractions from Extract Mu-1

**Figure 3:**
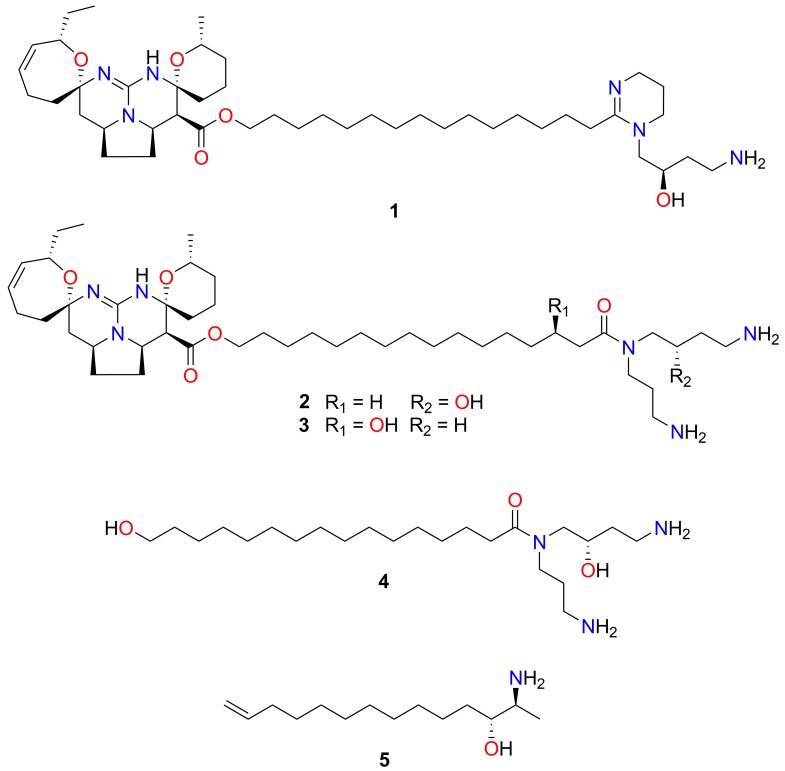
Chemical structures of the compounds purified in this study and other related alkaloids. Chemical strutures of fromiamycalin (**1**) purified from the extract of *Monanchora unguiculata* and other related pentacyclic guanidine alkaloids, crambescidin 800 (**2**), celeromycalin (**3**) as well as the hydroxyspermidine derivative (**4**) isolated in the original study of fromiamycalin purification [38], and halaminol A (**5**) purified from the extract of *Haliclona* sp.

Due to the significant reduction in L4 development by each of the three sequential fractions from the extract (*Mu*-1) of *M. unguiculata*, coupled to the availability of material from this taxon for further extraction and purification, extract *Mu*-1 was prioritised for the isolation of a chemically defined active compound. The tris-trifluoroacetate salt of the known guanidine alkaloid, fromiamycalin (**1**, 23.0 mg, 1.85% dry weight) (Figure 3), was identified in all active fractions of *Mu*-1 following comparison of NMR, MS and chiro-optical data with published values [38] (Figures S1, S2, S5 and S7). 

Fromiamycalin belongs to a unique family of complex pentacyclic guanidine alkaloids (PGAs) [38]. The hydrochloride salt of fromiamycalin (**1**), the compound purified in this study, was originally isolated from an acetone extract from the New Caledonian starfish, *Fromia monilis*, along with the related pentacyclic guanidine derivatives, crambescidin 800 (**2**), celeromycalin (**3**) and a hydroxyspermidine-hydroxyacid derivative (**4**) [38] (Figure 3); subsequently, **1** was also purified from *Monanchora arbuscula* [39]. Apart from fromiamycalin, several other PGAs, including ptilomycalins [40,41], monanchocidins [42,43], monanchomycalins [44,45], crambescidins 826, 359 and 431 [28,46] as well as normonanchocidins [47], have been purified from members of the genus *Monanchora*.

### 2.4. Fromiamycalin Inhibited the Motility and Development of Larvae of H. contortus, and had Moderate Toxicity on Fao Rat Hepatoma Cells

**Figure 4:**
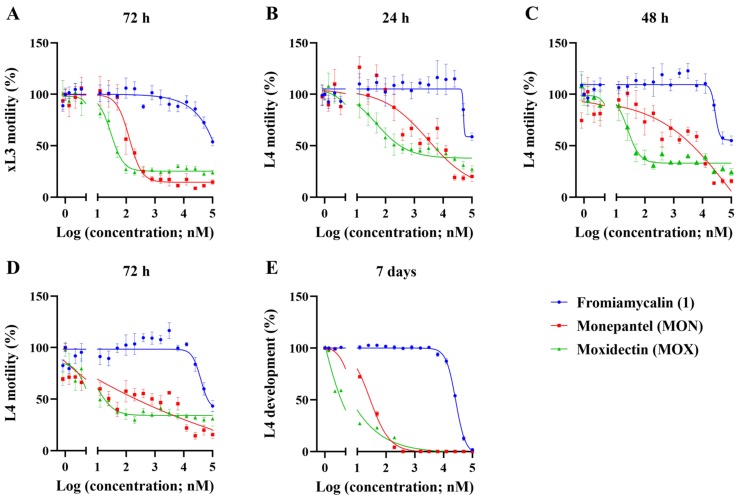
In vitro activity of fromiamycalin (1) on larval stages of *Haemonchus contortus*. Dose-response curves for fromiamycalin (**1**) on xL3 motility (panel **A**), L4 motility (panels **B**–**D**) and L4 development (panel **E**).

Fromiamycalin was shown to be a moderately potent inhibitor of L4 development (IC_50_ = 26.6 ± 0.74 µM, 7 days) and L4 motility (IC_50_ = 39.4 ± 4.83 µM, 72 h), although it was not potent at inhibiting xL3 motility (IC_50_ ≥ 100 µM, 72 h) of *H. contortus* (Table 2 and Figure 4). In previous studies, fromiamycalin exhibited anti-human immunodeficiency virus (HIV) activity (IC_50_ = 1–3 μM) [28] as well as activity against the malaria parasite, *Plasmodium falciparum,* with an IC_50_ of 0.24 μM [26]. Other compounds of the PGA family were reported also to have anti-malarial (ptilomycalin A and crambescidin 800 [39,48]), anti-viral (ptilomycalin A [49]) as well as anti-fungal (ptilomycalin A [49]) and/or anti-cancer (crambescidin 800 and celeromycalin [25,38]) activities. However, to the best of our knowledge, this is the first report of the activity of a member of this structural family on a socioeconomically important parasitic nematode (*H. contortus*) of animals. 

Fromiamycalin was tested for its toxicity on Fao rat hepatoma cells and had a low CC_50_ (50% cytotoxic concentration; this compound concentration reduces cell viability by 50%) value (5.7 ± 0.73 µM); it thus had limited selectivity (<1) for *H. contortus*. These results were consistent with a previous study [38], in which fromiamycalin (**1**) and crambescidin 800 (**2**) were reported to be moderately toxic to human peripheral blood CD4^+^ T lymphoblast (called CEM 4) cells infected by HIV-1, with a CC_50_ value of 0.11 μg/mL, whereas celeromycalin (**3**) was less cytotoxic, with a CC_50_ of 0.32 μg/mL. No cytoprotective effects were observed for any of these compounds (**1**–**3**) at a dose of < 0.1 μg/mL. Of note, the hydroxyspermidine derivative (**4**) exhibited weaker cytotoxicity with a CC_50_ value of 2.7 μg/mL, suggesting that the pentacyclic guanidine moiety is a significant contributor to cell toxicity [38].

### 2.5. NMR, MS and Chiro-optical Analyses Identified Halaminol A in the Active Fractions of Extracts Ha-1 and Ha-2

Separate ^1^H NMR and UPLC-MS analyses of the active fractions (fraction 43) of both *Ha*-1 and *Ha*-2, indicated that their chemical compositions were the same, but were comprised of a mixture of more than three amino alcohols, eluting from the column when the mobile phase reached 77% MeOH/H_2_O. These amino alcohols exhibited a lack of UV-absorbance, and poor chromatographic resolution due to significant tailing (~12 min on C_18_ stationary phase), despite the inclusion of mobile phase modifiers. Chromatographic purification was hindered by a significant overlap of ^1^H NMR characteristics (chemical shifts, couplings and multiplicities) among individual amino alcohols. Published data indicate that these compounds, particularly the less saturated analogues, are more amenable to separation as acetyl derivatives [50]. In our hands, attempts to acetylate (pyridine/Ac_2_O, 1:1, rt, 16 h) the active fractions (fraction 43) of *Ha*-1 and *Ha*-2 for chromatography resulted in a complex mixture with insufficient quantities of the expected reaction products to pursue structure elucidation and biological testing. The limited availability of *Haliclona* material precluded repeating the acetylation reaction on a larger scale. 

To ascertain the compound(s) responsible for the anthelmintic activity, a larger quantity of the amino alcohol mixture was obtained (173 mg, composed of fractions eluting between 65% and 78% MeOH/H_2_O) and further fractionated using reversed phased HPLC. ^1^H NMR analysis of the new fractions 40–60 indicated that the three major compounds of interest co-eluted across 14 fractions. The leading-fractions 47 and 48 contained small quantities of an inactive mixture of distinct halaminol derivatives (not identified), whilst the tailing-fractions 51–58 predominantly contained halaminol A (**5**). Using the same chromatographic conditions, the fraction enriched in halaminol A was subjected to two additional chromatography runs in order to obtain alkaloid **5** in high purity. The trifluoroacetate salt of the known compound, halaminol A (**5**, 3.8 mg, 99% purity) (Figure 3), was identified following comparison of NMR, MS and chiro-optical data with published values [50] (Figures S3, S4, S6 and S7).

### 2.6. Halaminol A Inhibited the Motility and/or Development of Larvae of H. contortus, and had Limited Toxicity on Fao Rat Hepatoma Cells

Halaminol A (**5**) was tested for its potency at inhibiting xL3 and L4 motility as well as L4 development. This compound was not a potent inhibitor of either motility or development of larvae and did not produce reliable dose-response curves when tested in two-fold dilution series, commencing at 100 µM. However, **5** exhibited a complete inhibition of L4 development at the highest tested concentration of 500 µM, and induced the same (i.e., ‘straight’) phenotype in xL3s as was observed for the extracts (*Ha*-1 and *Ha*-2) and active fractions (fraction 43), confirming that the purified compound is a significant, if not the main contributor, to the bioactivity of the source material. Moreover, ~ 50% inhibition of L4 motility (72 h) was observed at the highest tested concentration of 100 µM, and the treated L4s showed a ‘straight’ phenotype. Compound **5** had limited toxicity to Fao cells, with a CC_50_ value of 42.9 ± 2.56 µM. While halaminol A has known anti-fungal activity against *Trichophyton mentagrophytes* and *Cladosporium resinae* [50], and anti-fouling properties [51], there was no previous report of activity against any animal-parasitic nematode. Although the potency of compound 5 on *H. contortus* was relatively limited, extensive structure activity relationship (SAR) studies of the halaminol A scaffold are required to assess whether selectivity, activity and potency can be optimised.

## 3. Materials and Methods 

### 3.1. Marine Sponge Material: Extraction, Fractionation and Compound Characterisation

The marine invertebrate extract library (n = 2000) was sourced from NatureBank from GRIDD. The freeze-dried materials were processed at GRIDD to yield lead-like enhanced (LLE) extracts, as described previously [22], and solubilised in dimethyl sulfoxide (DMSO; Sigma Aldrich, St. Louis, MO, USA) to prepare a stock of 250 µge/µL for each extract. The concentration units, μge/μL, used for the NatureBank extract library relate to: (i) The amount of dry plant material that is weighed out for extraction and (ii) the amount of DMSO that the dry plant extract is dissolved in to make the extract for subsequent screening. For example, 300 mg equivalents (mge) is the extract derived from 300 mg of dry marine material; when this extract is dissolved in 1.2 mL of solvent (i.e., DMSO), the final stock solution concentration is 250 μge/μL. A detailed description of the method been published previously [22]. 

To undertake bioassay-guided fractionation of the extracts with biological activity against *H. contortus* (see Section 3.2), large-scale extracts were prepared, as described previously [52]. Briefly, the individual freeze-dried and ground marine invertebrate samples (~10 g) were sequentially extracted with *n*-hexane (250 mL, 2 h), CH_2_Cl_2_ (250 mL, 2 h) and MeOH (250 mL, 2 h; 250 mL, 16 h) on an orbital shaker (200 rpm; Bio-line, Edwards Instrument Company, Narellan, Australia). The *n*-hexane extracts were discarded, while the resultant MeOH and CH_2_Cl_2_ extracts were combined and dried under reduced pressure (MeOH/ CH_2_Cl_2_ extracts: 3.2 g of *Mu*-1, and 3.1 g and 3.2 g of *Ha*-1 and *Ha*-2, respectively).

A portion (400 mg) of each of the resultant extracts (i.e., *Mu*-1, *Ha*-1 and *Ha*-2) was separately pre-adsorbed to C_18_-bonded silica (Alltech C_18_, 35-75 µm, 150 Å), packed into a stainless-steel guard cartridge (Alltech, 10 × 30 mm), and the assembled column coupled to a Betasil C_18_ column (5 µm, 143 Å, 21.2 × 150 mm, Thermo Fisher, Waltham, MA, USA). The fractionation was performed using a mobile phase consisting of isocratic conditions of H_2_O/MeOH/TFA (90:10:0.1) for the first 10 min, followed by a linear gradient to MeOH/TFA (100:0.1) over 40 min, then isocratic conditions of MeOH/TFA (100:0.1) for a further 10 min, all at a flow rate of 9 mL/min. From each of the three extracts, 60 fractions were collected over time (60 × 1 min). A Waters 600 pump, 996 photodiode array detector and a Gilson 215 liquid handler were used for the HPLC experiments (Gilson, Middleton, WI, USA).

The active fractions of *Mu*-1 were analysed by ^1^H NMR spectroscopy and UPLC-MS to evaluate their chemical composition and purity. Spectroscopic and spectrometric analyses and comparison of data with the published literature identified one major compound in all bioactive fractions. 

Attempts to acetylate the active fractions of *Ha*-1 and *Ha*-2 resulted in a complex mixtures and low yields of key derivatives. Therefore, the remaining extract of *Ha*-1 (~2.2 g) was fractionated by repeated semi-preparative HPLC, as outlined above to obtain an enriched fraction of amino alcohols (173 mg) by combining fractions eluting in the range of 65–78% MeOH/H_2_O. This mixture of amino alcohols was further fractionated using isocratic conditions of H_2_O/MeOH/TFA (70:30:0.1) for the first 1 min, followed by a linear gradient to H_2_O/MeOH/TFA (50:50:0.1) over 9 min, a linear gradient to H_2_O/MeOH/TFA (25:75:0.1) over 70 min, then a linear gradient to H_2_O/MeOH/TFA (5:95:0.1) over 5 min, and lastly, isocratic conditions of H_2_O/MeOH/TFA (5:95:0.1) for a further 5 min, all at a flow rate of 9 ml/min. Fractions 40 to 60 were analysed by ^1^H NMR, and repeated chromatographic separation of the leading and tailing fractions of this region was undertaken to obtain a minor mixture of (unidentified) lipids and compound **5** in sufficient quantities and purity to proceed with the bioassay and structural confirmation.

Specific optical rotations were recorded using a JASCO P-1020 polarimeter (Easton, MD, USA), and NMR spectra were recorded on a Bruker AVANCE HDX 800 MHz NMR spectrometer (Fällanden, Zürich, Switzerland). MestReNova v.11.0 software (Santiago de Compostela, Galicia, Spain) was used to process the NMR spectra, and the ^1^H and ^13^C NMR chemical shifts were referenced to the solvent peaks for CDCl_3_ at *δ*_H_ 7.26 and *δ*_C_ 77.16, and CD_3_OD at *δ*_H_ 3.31 and *δ*_C_ 49.00. A Thermo Scientific Ultimate 3000 UPLC-MS spectrometer fitted with an Acquity CSH C_18_ column (1.7 µm 130 Å, 2.1 × 150 mm, Waters, Milford, MA, USA) was used for low resolution electrospray UPLC-MS analyses. All of the solvents used for extraction and chromatography, and NMR and MS analyses were HPLC grade and purchased from Honeywell Burdick & Jackson (Muskegon, MI, USA), except for trifluoroacetic acid (TFA, Sigma-Aldrich, St. Louis, MO, USA).

### 3.2. Procurement of H. contortus, and Bioassays to Identify Active Extracts and Chromatographic Fractions

*H. contortus* (Haecon-5 strain) was maintained in experimental sheep, as described previously [53]. In brief, individual sheep were infected with 7000 third-stage larvae (L3s) of *H. contortus*, in accordance with strict animal ethics guidelines (permit no. 1413429; The University of Melbourne). Faeces were collected and incubated for seven days at 27 °C, to allow first-stage larvae (L1s) to emerge and develop to L3s, which were then collected and stored at 11 °C until used. The xL3s were produced by incubating L3s in 0.15% sodium hypochlorite (NaClO) for 20 min at 37 °C, and L4s were produced in culture by incubating xL3s at 10% v/v CO_2_ at 38 °C for seven days [53]. 

Individual LLE extracts were screened for their inhibitory effects on xL3 motility using an established protocol [53]. The extracts were diluted to a final concentration of 3 µge/µL (+1.25% DMSO) in Luria Bertani (LB) medium supplemented with antibiotic-antimycotic agent (Gibco, Gaithersburg, MD, USA) (LB*) and dispensed into 96-well flat-bottom plates. Then, xL3s were dispensed into individual wells at a density of 300 larvae per well in 50 µL of LB*. Monepantel (Zolvix, Elanco Animal Health, Sydney, Australia) and moxidectin (Cydectin, Virbac, Carros, France) (20 µM) were used as positive-control compounds, and LB* + 1.25% DMSO as the negative (untreated) -controls. The plates were incubated at 38 °C in a CO_2_ (10% v/v) incubator for 72 h, and a 5 s-video recording was acquired from each well and processed using a video-graphic image analysis approach [53]. A motility index (Mi) was obtained for each well, as described previously [53]. Extracts that reduced xL3 motility by ≥ 70% (calculated using the program GraphPad Prism v.7.02 software) were considered as active (i.e., a ‘hit’) and used to direct fractionation efforts.

HPLC fractions (n = 60) collected from each active extract were individually tested (at 3 µge/µL + 1.25% DMSO) for inhibitory effects on the motility of xL3s (as explained above in this subsection) and the development of xL3s into L4s (i.e., L4 development) [53]. To assess L4 development, xL3s were treated with fractions, and the number/percentage of xL3s that developed into L4s (identified based on their well-developed mouth and pharynx [54]) was counted/calculated following an incubation for seven days [53]. The data were analysed using non-parametric one-way ANOVA and Dunnett’s multiple comparison tests (GraphPad Prism v.7.02 software). The fractions with significant inhibitory effects on xL3 motility and/or L4 development were selected for compound purification.

### 3.3. Assessment of the Potency of Extracts and Purified Compounds

The potencies of active LLE extracts to inhibit xL3 motility and L4 development were assessed by testing them in an 18-point two-fold dilution series, commencing at 3 µge/µL (+ 1.25% DMSO). Similarly, the purified compounds were tested in a two-fold dilution series, starting at 100 µM (+ 0.5% DMSO), for their inhibitory effect on xL3 or L4 motility at 24 h, 48 h and 72 h and on L4 development at day seven (cf. Section 3.2). Compound **5** was further tested at 500 µM, 400 µM, 300 µM and 200 µM for its inhibitory effect on L4 development. For all experiments, matched concentrations of monepantel and moxidectin, and LB* with matched percentages of DMSO were used as test-positive and -negative (untreated) controls, respectively. The maximum concentration of DMSO that the larvae were exposed to was 1.25% (for extracts) or 0.5% (for purified compounds) [53]. All motility and development assays, with three technical replicates in each assay, were repeated three times. The half-maximum inhibitory concentration (IC_50_) of each extract or compound was determined by transforming the concentration to log_10_ and fitting to a variable slope four-parameter model using GraphPad software (Prism v.7.02; San Diego, CA, USA) [53].

### 3.4. Assessment of Cytotoxicity of Purified Compounds

The purified compounds were tested in a two-fold dilution series (nine points, commencing at a concentration that achieved 100% inhibition of L4 development) for their toxicities to Fao rat hepatoma cells, as described previously [55]. In brief, Fao cells in RPMI-1640 medium (Gibco, Gaithersburg, MD, USA) supplemented with 10% foetal bovine serum (AusGenex, Molendinar, Australia) were seeded in 96-well flat-bottom well plates (50,000 cells per well) and treated with individual compounds or controls. Monepantel and moxidectin (larval activity; commencing at 100 µM) and camptothecin (cell toxicity; 5 µM) were used as positive-controls, and the matched DMSO concentrations were used as negative (untreated) controls. The treated cells were then incubated for 48 h at 37 °C with 5% v/v CO_2_. At 48 h, the cells were washed once with 100 µL of phosphate-buffered saline (PBS). The cells were then stained with 40 µL of 0.5 % crystal violet staining solution (Sigma-Aldrich, St. Louis, MO, USA) in 30% ethanol for 10 min at room temperature. Subsequently, the cells were washed three times with 200 µL of PBS and lysed with 200 µL of 1% sodium dodecyl sulfate (SDS). The optical density of the suspension in each well was measured at 595 nm using a plate reader. The CC_50_ values were calculated using the same method as used for the calculation of IC_50_ values (see Section 3.3). The selectivity index (SI) for *H. contortus* was calculated by dividing the CC_50_ value for Fao cells by the IC_50_ value for *H. contortus* [53].

## 4. Conclusions

Here, we screened a collection of 2000 extracts from Australian marine invertebrates for biological activity against the economically important parasitic nematode *H. contortus*. The primary screen identified three extracts with anthelmintic activity, namely *Mu*-1 from *M. unguiculata*, and *Ha*-1 and *Ha*-2 from *Haliclona* sp. Each of the extracts inhibited both motility and development of larvae of *H. contortus*. Bioassay-guided fractionation of each extract revealed that activity against *H. contortus* was limited to one to three chromatographic fractions, and led to subsequent identification of fromiamycalin, a previously reported PGA, from the extract *Mu*-1. Fromiamycalin was moderately potent at inhibiting L4 motility and L4 development of *H. contortus*. Halaminol A was identified as the major compound in the active fractions of extracts *Ha*-1 and *Ha*-2, and it had limited potency at inhibiting xL3 and L4 motility, and L4 development. In conclusion, this is the first report of the activity of both fromiamycalin (and, indeed, any PGA representative) and halaminol A against an animal-parasitic nematode. The results of this study encourage future medicinal/natural product chemistry studies to develop fromiamycalin, related PGA compounds and halaminols as potential anthelmintic candidates.

## Figures and Tables

**Table 1:** In vitro activity of three marine sponge extracts. Comparison of the half-maximum inhibitory concentration (IC_50_) values for the inhibitory effects of the marine sponge extracts on exsheathed third-stage larvae (xL3s) motility or fourth-stage larvae (L4) development of *Haemonchus contortus*. IC_50_ values are expressed as a mean IC_50_ ± standard error of mean. Corresponding values for monepantel and moxidectin (controls) are included for comparison.

Extract Control Compound	xL3 Motility (IC_50_ in µge/µL)	L4 Development (IC_50_ in µge/µL)
	72 h	7 days
***Monanchora unguiculata*** **(*****Mu*****-1)**	0.7 ± 0.01	0.6 ± 0.17
***Haliclona* sp. (*Ha*-1)**	~ 3.0 ^a^	0.6 ± 0.18
***Haliclona* sp. (*Ha*-2)**	~ 3.0 ^a^	0.6 ± 0.15
**Monepantel ^b^**	6.6 10^−5^ ± 0.06	1.4 10^−5^ ± 0.00
**Moxidectin ^b^**	1.9 10^−5^ ± 0.06	2.6 10^−6^ ± 0.00

^a^ IC_50_ values estimated from graphs in Figure 1.; ^b^ IC_50_ values given as μg/μL.

**Table 2:** In vitro activity of fromiamycalin. Half-maximum inhibitory concentration (IC_50_) values for the inhibitory effects of fromiamycalin on the xL3 and L4 motility, and on L4 development of *Haemonchus contortus*. IC_50_ values are expressed as a mean IC_50_ ± standard error of mean or a range of values when a defined curve could not be fitted to the data. Corresponding values for monepantel and moxidectin (controls) are included for comparison.

Test Compound Control Compound	xL3 Motility (IC_50_ in µM)	L4 Motility (IC_50_ in µM)			L4 Development (IC_50_ in µM)
	72 h	24 h	48 h	72 h	7 days
**Fromiamycalin**	> 100	52.4 ± 3.34	31.9 ± 3.74	39.4 ± 4.83	26.6 ± 0.74
**Monepantel**	0.1 ± 0.06	1.6 ± 0.73	0.2 ± 0.00	0.1–0.2 ^a^	0.03 ± 0.00
**Moxidectin**	0.03 ± 0.06	0.06 ± 0.05	0.04 ± 0.02	0.008 ^a^	0.004 ± 0.00

^a^ IC_50_ values estimated from graphs in Figure 3.

## Supplementary Materials

The following are available online at https://www.mdpi.com/1660-3397/17/11/598/s1, Figures S1–S7: ^1^H NMR, ^13^C NMR, MS and [α]D the TFA salts of fromiamycalin (**1**) and halaminol A (**5**).
