# Supplementary material for: Identification of Fromiamycalin and Halaminol A from Australian Marine Sponge Extracts with Anthelmintic Activity against Haemonchus contortus
Source: Mar Drugs. 2019 Oct 23;17(11):598. doi: 10.3390/md17110598 (PMC6891614; doi:10.3390/md17110598)
Supplement: Supplementary file 1 [file marinedrugs-17-00598-s001.pdf]

Supplementary Materials Contents Page

Identification of Fromiamycalin and Halaminol A from Australian Marine Sponge Extracts with Anthelmintic Activity against *Haemonchus contortus*

H. M. P. Dilrukshi Herath [1], Sarah Preston [1,2], Abdul Jabbar [1], Jose Garcia-Bustos [1], Aya C. Taki [1], Russell S. Addison [3], Sasha Hayes [3], Karren D. Beattie [3], Sean L. McGee [4], Sheree Martin [4], Merrick G. Ekins [5], John N. A. Hooper [5], Bill C. H. Chang [1], Andreas Hofmann [1,3], Rohan A. Davis [3,*], Robin B. Gasser [1,*]

1 Faculty of Veterinary and Agricultural Sciences, The University of Melbourne, Parkville, Victoria 3010, Australia; E-mail: herathh@student.unimelb.edu.au (H.M.P.D.H); sarah.jm.preston@gmail.com (S.P); jabbara@unimelb.edu.au (A.J); Jose.GarciaB@unimelb.edu.au (J.G.B); aya.taki@unimelb.edu.au (A.C.T); bchang@yourgene.com.tw (B.C.H.C); a.hofmann@structuralchemistry.org (A.H); robinbg@unimelb.edu.au (R.B.G)
2 Faculty of Science and Technology, Federation University, Ballarat, Victoria 3350, Australia; E-mail: sarah.jm.preston@gmail.com (S.P)
3 Griffith Institute for Drug Discovery, Griffith University, Brisbane, QLD 4111, Australia; E-mail: r.addison@griffith.edu.au (R.S.A); sasha.hayes@griffith.edu.au (S.H); a.hofmann@structuralchemistry.org (A.H); k.beattie@griffith.edu.au (K.D.B); r.davis@griffith.edu.au (R.A.D)
4 Metabolic Research Unit, Metabolic Reprogramming Laboratory, School of Medicine, Faculty of Health, Deakin University, Waurn Ponds, Victoria 3216, Australia; E-mail: sean.mcgee@deakin.edu.au (S.L.M), sheree.martin@deakin.edu.au (S.M)
5 Queensland Museum, South Brisbane BC, QLD 4101, Australia; E-mail: merrick.ekins@qm.qld.gov.au (M.G.E); john.hooper@qm.qld.gov.au (J.N.A.H)

***** Correspondence: robinbg@unimelb.edu.au (R.B.G); Tel: +61-3-9731-2283. Fax: +61-3-9731-2366; r.davis@griffith.edu.au (R.A.D); Tel: +61-7-3735-6043. Fax: +61-7-3735-6001

Contents:

Figure S1 ^{1}H NMR (800 MHz) spectrum of fromiamycalin tris-TFA salt (**1**) in CD_3OD
Figure S2 ^{13}C NMR (200 MHz) spectrum of fromiamycalin tris-TFA salt (**1**) in CD_3OD
Figure S3 ^{1}H NMR (800 MHz) spectrum of halaminol A TFA salt (**5**) in CD_3OD
Figure S4 ^{13}C NMR (200 MHz) spectrum of halaminol A TFA salt (**5**) in CD_3OD
Figure S5 (+)-LRESIMS of fromiamycalin tris-TFA salt (**1**)
Figure S6 (+)-LRESIMS of halaminol A TFA salt (**5**)
Figure S7 Other experimental data for fromiamycalin tris-TFA salt (**1**) and halaminol A TFA salt (**5**)

Figure S1 ^{1}H NMR (800 MHz) spectrum of fromiamycalin tris-TFA salt (**1**) in CD_3OD

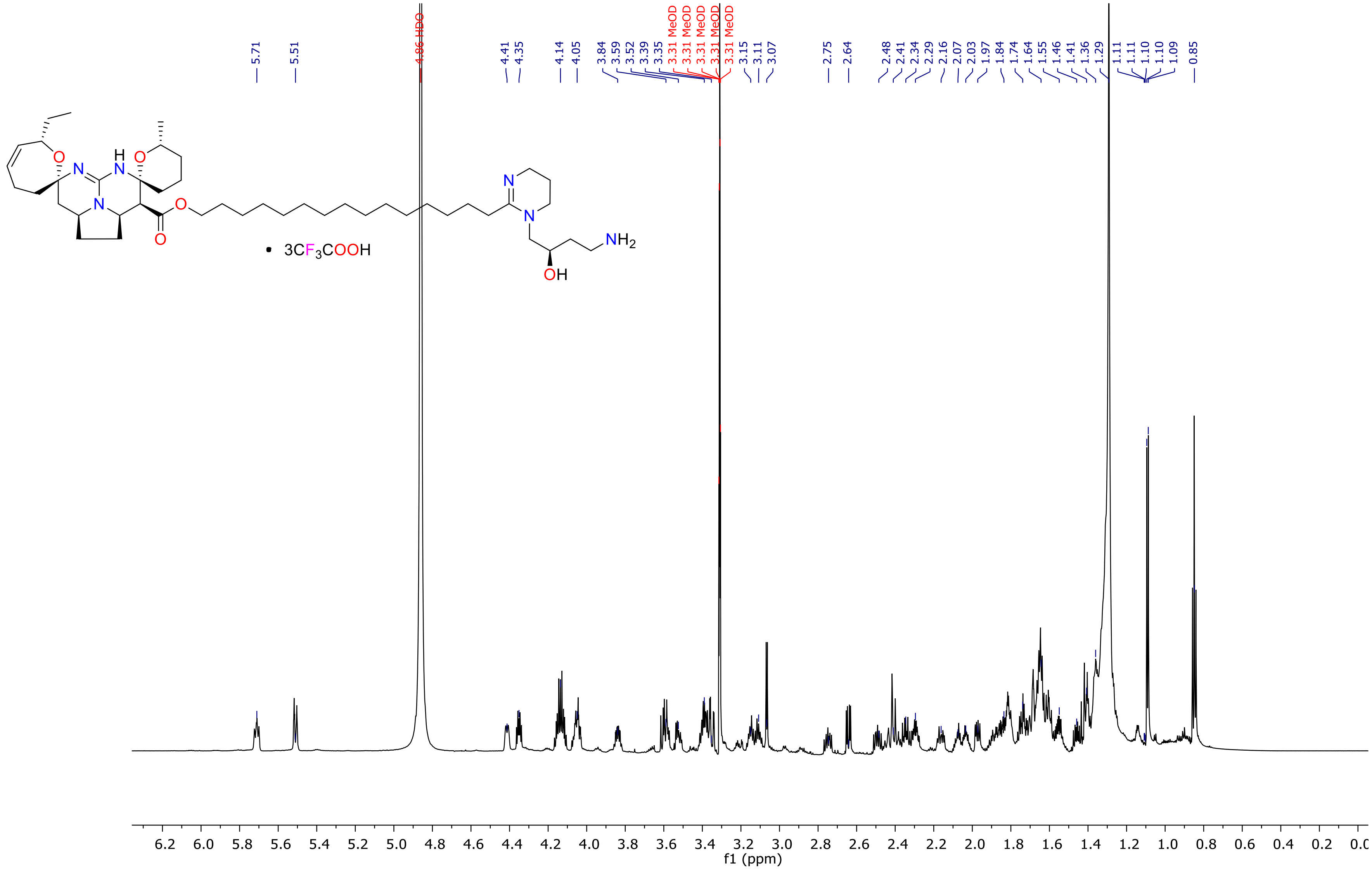

Figure S2 ^{13}C NMR (200 MHz) spectrum of fromiamycalin tris-TFA salt (**1**) in CD_3OD

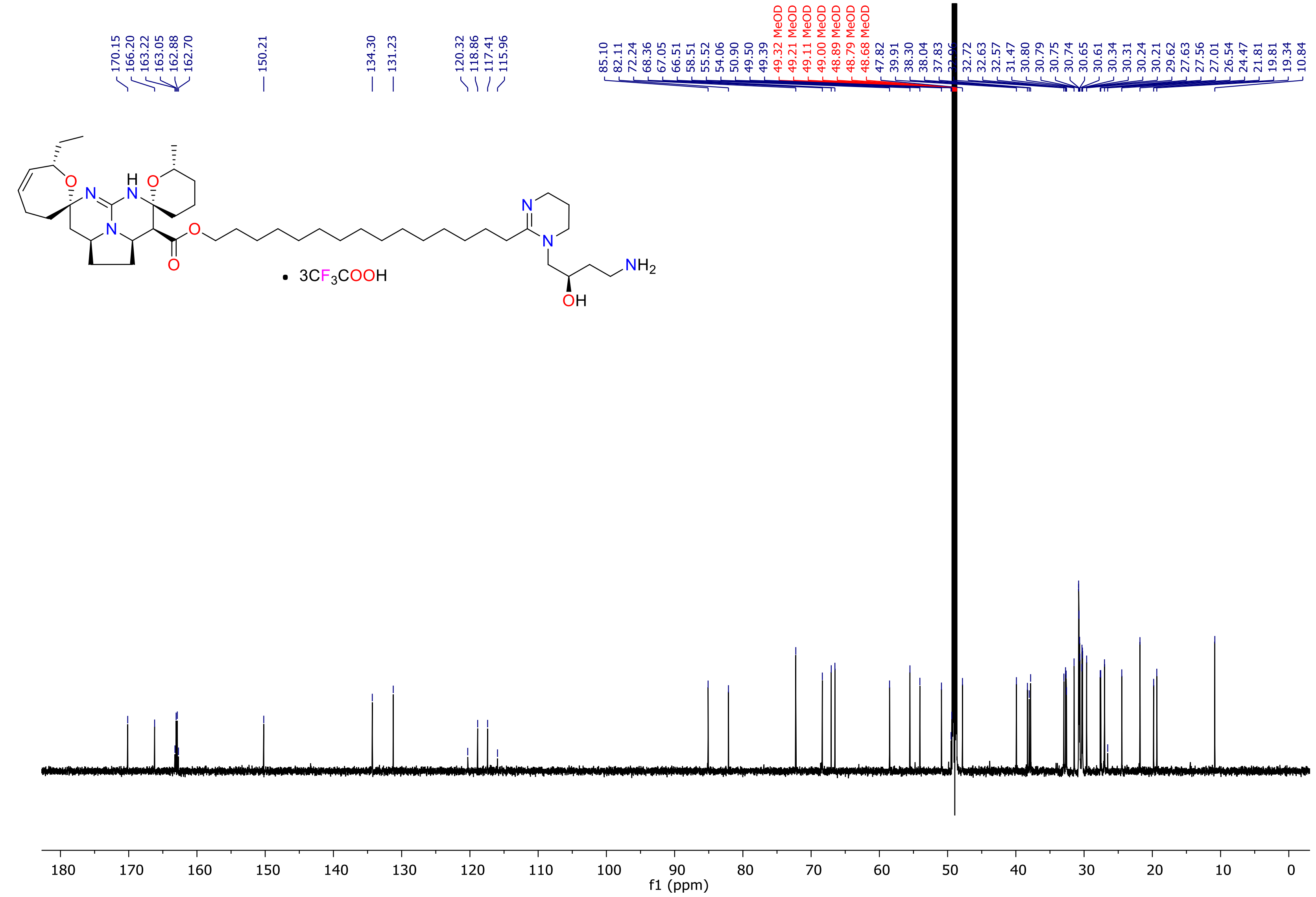

Figure S3 ^{1}H NMR (800 MHz) spectrum of halaminol A TFA salt (**5**) in CD_3OD

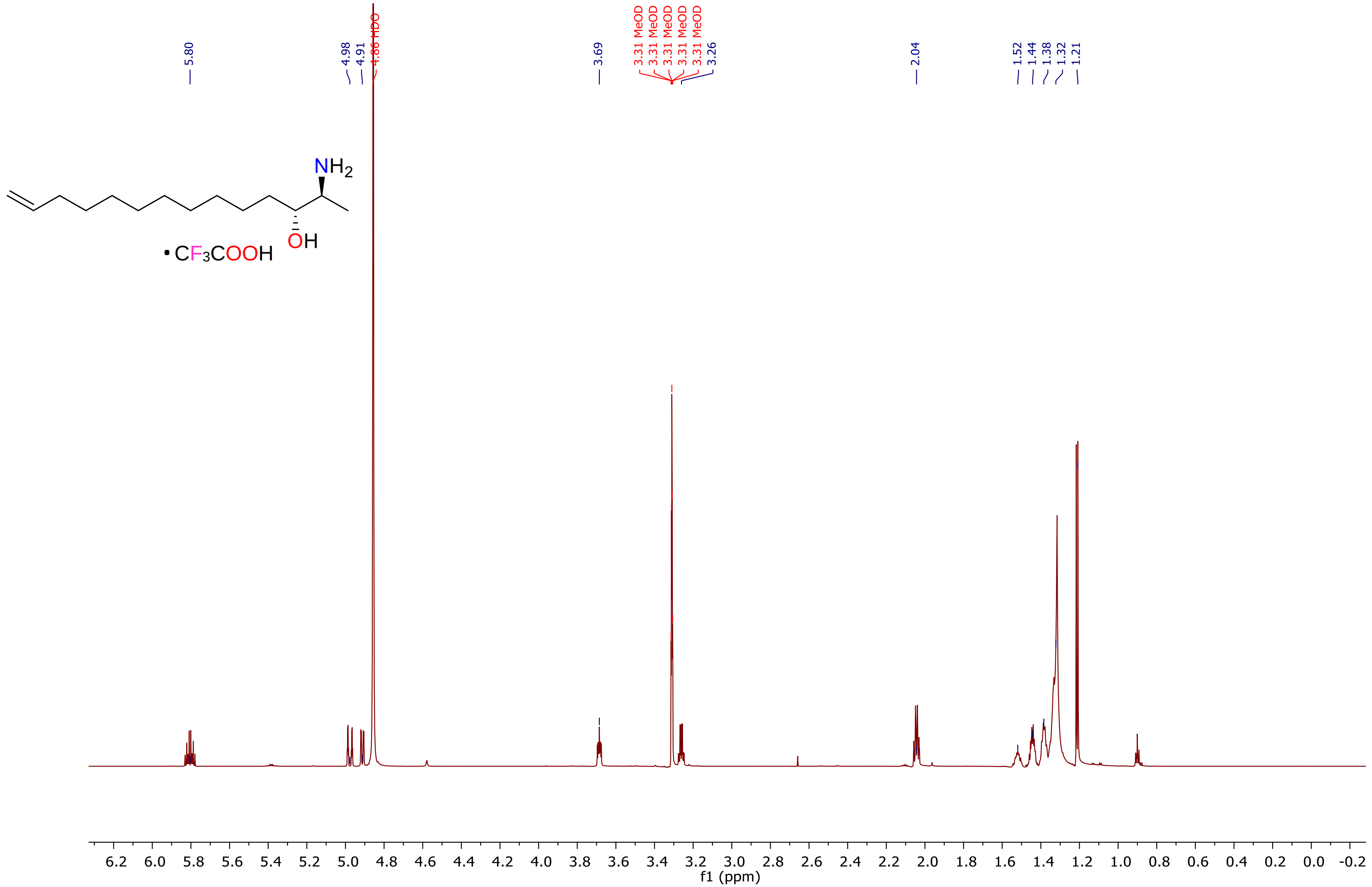

Figure S4 ^{13}C NMR (200 MHz) spectrum of halaminol A TFA salt (**5**) in CD_3OD

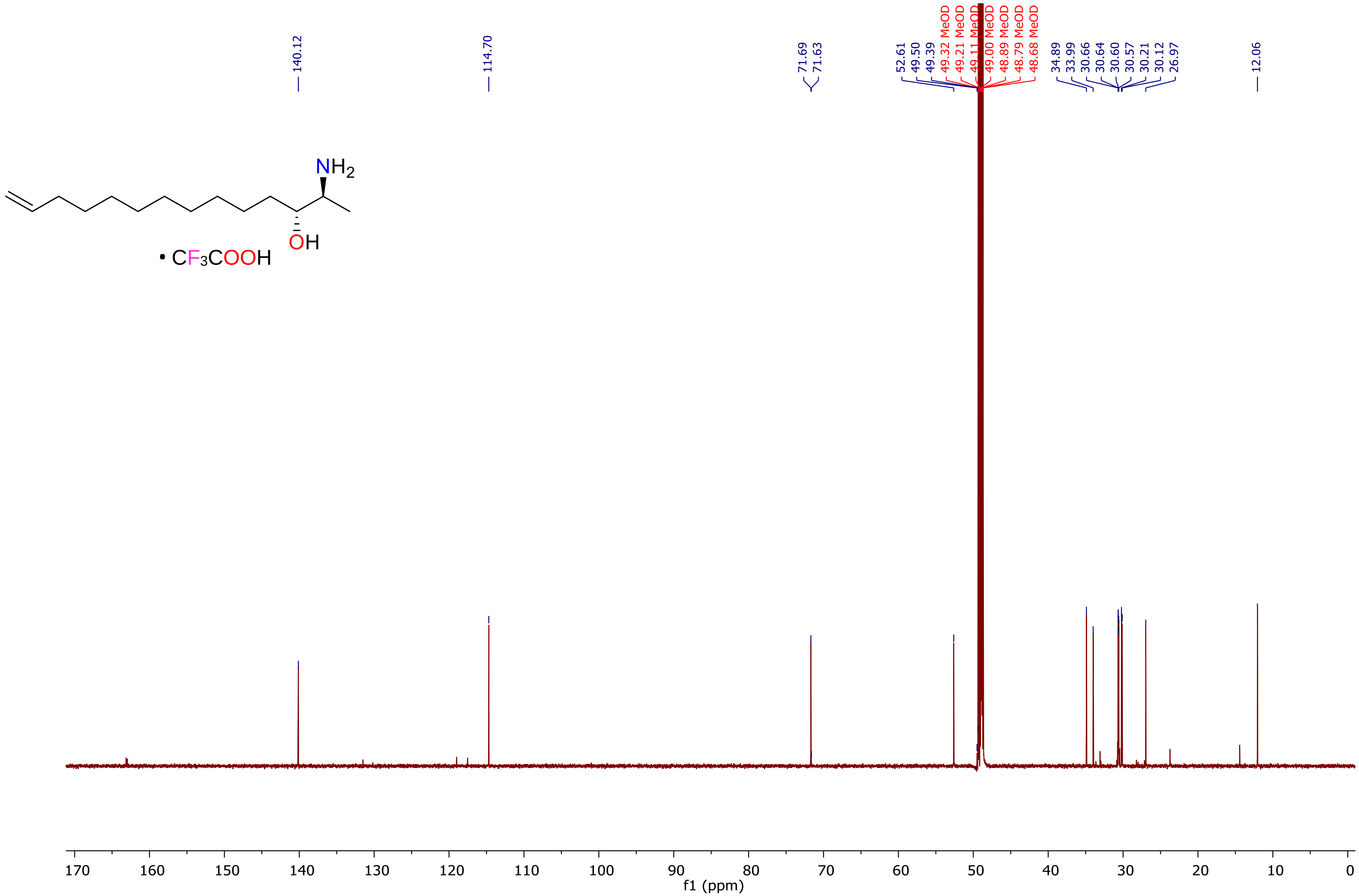

Figure S5 (+)-LRESIMS of fromiamycalin tris-TFA salt (**1**)

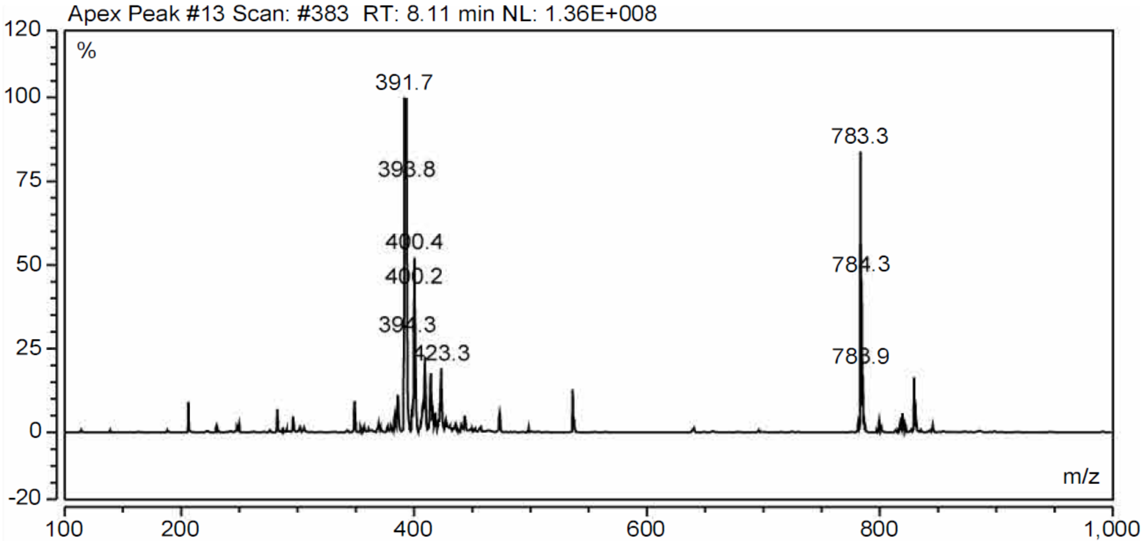

Figure S6 (+)-LRESIMS of halaminol A TFA salt (**5**)

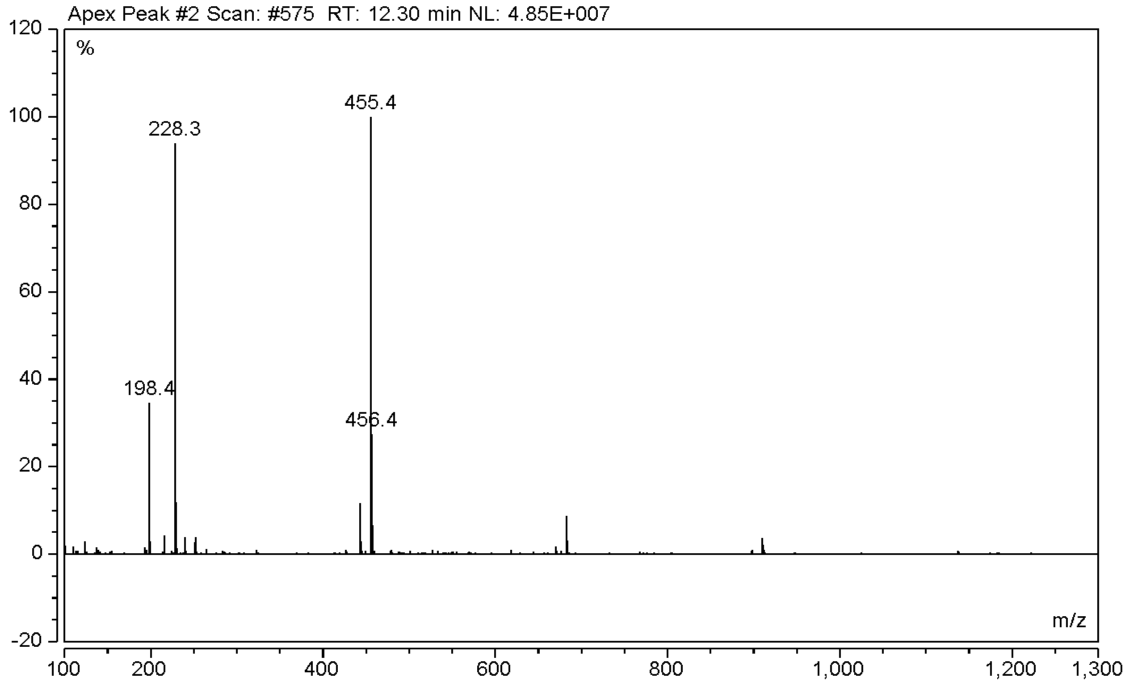

Figure S7 Other experimental data for fromiamycalin tris-TFA salt (**1**) and halaminol A TFA salt (**5**)

*Fromiamycalin tris TFA salt (**1**):* clear film; $[\alpha]_D^{25}$-15.4 (*c* 0.028, MeOH); lit. $[\alpha]_D$-12 (concentration and solvent not specified in the original paper [1]); (+)-LRESIMS *m/z* 392 (100), 784 (80) $[M+H]^+$.

*Halaminol A TFA salt (**5**):* clear oil; $[\alpha]_D^{24}$ +2.0 (*c* 0.049, CH_2Cl_2); lit. $[\alpha]_D$ +1.7 (*c* 0.044, CH_2Cl_2) [2]); (+)-LRESIMS *m/z* 228 (95) $[M+H]^+$, 455 (100) $[2M+H]^+$.

Note: The ^{13}C NMR data for **5** was consistent with that previously published by Clark et al. [2] with the exception of the C-5 chemical shift, which was originally reported as δ_C 30.6. This original assignment for C-5 appears to be a typographical error; we have revised the chemical shift for C-5 to δ_C 27.0, based on our analysis of the 1D/2D NMR data.

References:

1. Palagiano, E.; de Marino, S.; Minale, L.; Riccio, R.; Zollo, F.; Iorizzi, M.; Carre, J.B.; Debitus, C.; Lucarain, L.; Provost, J. Ptilomycalin A, crambescidin 800 and related new highly cytotoxic guanidine alkaloids from the starfishes *Fromia monilis* and *Celerina heffernani*. *Tetrahedron*. **1995**, *51*, 3675-3682.

2. Clark, R.J.; Garson, M.J.; Hooper, J.N.A. Antifungal alkyl amino alcohols from the tropical marine sponge *Haliclona* n. sp. *J. Nat. Prod*. **2001**, *64*, 1568-1571.
